# Change over Five Years in Important Measures of Methodological Quality and Reporting in Randomized Cardiovascular Clinical Trials

**DOI:** 10.3390/jcdd11010002

**Published:** 2023-12-21

**Authors:** Odgerel Baasan, Omar Freihat, Dávid U. Nagy, Szimonetta Lohner

**Affiliations:** 1 Doctoral School of Health Sciences, University of Pécs, 7621 Pécs, Hungary; 2 Cochrane Hungary, Clinical Centre of the University of Pécs, Medical School, University of Pécs, 7624 Pécs, Hungary; 3 Institute of Geobotany/Plant Ecology, Martin-Luther-University, 06108 Halle, Germany; 4 Department of Public Health Medicine, Medical School, University of Pécs, 7624 Pécs, Hungary

**Keywords:** cardiovascular disease, randomized clinical trials, risk of bias, trial registration, data monitoring committee

## Abstract

Objectives: The aim of our current study was to analyze whether the use of important measures of methodological quality and reporting of randomized clinical trials published in the field of cardiovascular disease research haschanged over time. A furtheraim was to investigate whether there was an improvement over time in the ability of these trials to provide a good estimate of the true intervention effect. Methods: We conducted two searches in the Cochrane Central Register of Controlled Trials (CENTAL) database to identify randomized cardiovascular clinical trials published in either 2012 or 2017. Randomized clinical trials (RCTs) trials in cardiovascular disease research with adult participants were eligible to be included. We randomly selected 250 RCTs for publication years 2012 and 2017. Trial characteristics, data on measures of methodological quality, and reporting were extracted and the risk of bias for each trial was assessed. Results: As compared to 2012, in 2017 there were significant improvements in the reporting of the presence of a data monitoring committee (42.0% in 2017 compared to 34.4% in 2012; *p* < 0.001), and a positive change in registering randomized cardiovascular disease research in clinical trial registries (78.4% in 2017 compared to 68.9% in 2012; *p* = 0.03). We also observed that significantly more RCTs reported sample size calculation (60.4% in 2017 compared to 49.6% in 2012; *p* < 0.01) in 2017 as compared to 2012. RCTs in 2017 were more likely to have a low overall risk of bias (RoB) than in 2012 (29.2% in 2017 compared to 21.2% in 2012; *p* < 0.01). However, fewer 2017 RCTs were rated low (50.8% compared to 65.6%; *p* < 0.001) risk for blinding of participants and personnel, for blinding of outcome assessors (82.4% compared to 90.8%; *p* < 0.001), and selective outcome reporting (62.8% compared to 80.0%; <0.001). Conclusions: As compared to 2012, in 2017 there were significant improvements in some, but not all, the important measures of methodological quality. Although more trials in the field of cardiovascular disease research had a lower overall RoB in 2017, the improvement over time was not consistently perceived in all RoB domains.

## 1. Introduction

Randomized clinical trials (RCTs) constitute the foundational background of modern medical practice [1]. In the last three decades, the cardiovascular randomized clinical trial has emerged as the principal method by which new therapies are evaluated [2]. Moreover, evidence generated from randomized clinical trials has greatly influenced the diagnosis and treatment of many heart diseases including arterial hypertension, arrhythmias, acute myocardial infarction, heart failure, and coronary revascularization [3,4,5].

The increasing prevalence of cardiovascular disease around the world requires high-quality of clinical research and translation of its findings into new therapeutic and diagnostic strategies [6].

Unfortunately, although there was a significant increase in the quantity of scientific literature concerning cardiovascular disease published in recent years, it was indicated that this has not resulted in guideline recommendations with more certainty and supporting evidence. The American College of Cardiology and the American Heart Association (ACC/AHA) clinical practice guidelines are still based on a lower quality of evidence and expert opinions, indicating the lack of high-quality studies with relevant data [7].

Several tools exist that support researchers to plan and conduct high-quality research and make trial results completely and transparently available. Guidelines for clinical trial protocols (e.g., SPIRIT) facilitate trial planning in all important details. Reporting guidelines (e.g., CONSORT for RCTs) have the aim of decreasing the risk of non-reporting bias, i.e., facilitating that clinical trial methods are described as they were conducted and trial results are fully published [8]. The requirement of clinical trial registration supports transparency in research. In the USA it is a requirement from the Food and Drug Administration (FDA) that all clinical trials are registered before the first patient is enrolled [9], and the European Medicines Agency and WHO also support clinical trial registration [10,11]. In the field of cardiology, insufficient registration tendencies were reported [12]. Cardiac and cardiovascular system journals infrequently require, recommend, and enforce the use of obligatory clinical trial registration [13].

Methodological flaws in the design, conduct, analysis, and reporting of randomized clinical trials can cause the true intervention effect to be underestimated or overestimated. This is why these systemic errors (defined as the risk of bias) are assessed when systematic reviews are conducted or evidence-based guidelines are developed [14]. Concerns arising due to the high risk of bias in trials included in evidence syntheses lead to the downgrading of evidence level and consequently will decrease our certainty in the pooled results.

Our previous study compared the risk of bias in industry-funded and non-industry-funded cardiovascular disease research trials published in 2017 [15]. The present study investigated tendencies over time to answer whether there was an improvement in measures of methodological quality and reporting in randomized cardiovascular clinical trials between 2012 and 2017. Further, assessed how well these trials were able to estimate the true intervention effect in 2017 as compared to 2012.

## 2. Methods

We conducted two searches to identify randomized cardiovascular clinical trials published in either 2012 or 2017. We searched the Cochrane Central Register of Controlled Trials (CENTAL) database, as this is the most comprehensive resource available containing randomized clinical trials.

We used the same search strategy for both years, containing subject headings and keywords related to adults (aged > 18 years) and cardiovascular diseases, restricted to the years 2012 or 2017. The first author (OB) searched CENTRAL and screening trials for eligibility. We included studies that were published in 2012 in English language journals, where the investigated intervention was related to cardiovascular practice, and where participants 18 years or older were included. Our search resulted in 2566 trials. All identified records were exported to Excel, where they were randomly ordered. In a subsequent step, we were reordering trials from the smallest to the highest number.

We included the first 250 (about 10%) eligible randomized clinical trials for both year 2012 and 2017.

We used a data extraction tool that was developed for assessing the methodological quality of RCTs in child health research [15]. Two authors (OB, OF) independently extracted data for each study included. We discussed all unclear decisions until a consensus was established. For each cardiovascular disease study, we identified information about journal type (e.g., general or specialty medical journal and general or specialty cardiovascular journal), corresponding author’s country, study type, study design, intervention type, type of control, number of study centers, study sample, primary diagnostic category in the study using the ICD-10 classification system, presence of data monitoring committee, type of primary outcome, outcome results, and trial registration. Trial registration numbers, author names, and keywords related to the specific cardiovascular intervention were used to find the published protocol via Google and Google Scholar. We completed data extraction after a precise analysis of full-text articles, trial registration, and published protocols. To retrieve information on the primary outcome of the trial, we defined primary outcome as (1) the outcome defined under the objective of the study, (2) the outcome used to calculate sample size, or (3) the first outcome reported in the randomized clinical trial.

We used the Cochrane RoB tool [16] to assess the methodological quality of randomized clinical trials. This tool evaluates 7 domains of bias and thereby determines the extent to which the RCT’s design, conduct, analysis, and presentation were appropriate to answer the trial research question. These 7 domains are (1) sequence generation (whether the allocation sequence was adequately generated), (2) allocation concealment (whether the allocation of group assignment could not been foreseen prior to randomization), (3) blinding of participants and personnel (whether the knowledge of the allocated intervention was adequately prevented during study), (4) blinding of outcome assessors, (5) incomplete outcome data (whether the incomplete outcome data were adequately addressed), (6) selective outcome reporting (whether the study was free of apparent selective outcome reporting), and (7) other sources of bias (whether the study was free of other problems that could introduce bias).

### Statistical Analysis

Statistical analyses were conducted by the statistical software R version 4.1.2 (R Development Core Team 2021) [17]. To analyze for 5-year changes in main study characteristics, we compared the 2017 sample with 250 RCTs published in 2012 [15]. All collected binomial variables were analyzed using logistic regression analyses with generalized linear models. All collected categorical variables with more than two categories were analyzed with multinomial regression models. For these models, a single *p*-value for the entire model was presented to provide a concise overview of the overall significance. Variables of methodological quality and report were analyzed with separate univariable logistic regressions.

Model assumptions on residuals were checked using “model-checking plots”. Statistical significance tests in the models were carried out with Chi-square tests. The value of *p* < 0.05 was considered as a significant result.

## 3. Results

### 3.1. Descriptive Analysis

The main characteristics of included cardiovascular RCTs are shown in Table 1. Data from 2017 have been previously partly reported [15]. Values from 2012, some data on additional measures of methodological quality and reporting for both years and statistical comparisons were novel.

We observed significant differences in the country of origin defined based on the first author’s affiliation between 2012 and 2017. In our 2017 sample, more publications were published in specialty medical journals (19.6% compared to 10.4%; the logistic regression result on the Type of Journal variable was: *p* < 0.001). In 2017 we included more RCTs with parallel design (92.4% compared to 80.4%; *p* < 0.01), and among the interventions there were more drug trials (55.6% compared to 46.8%) and surgical interventions (1.2% compared to 0.4%), (*p* < 0.001). In the 2017 sample, we had a larger number of multinational trials (27.6% compared to 18%), (*p* < 0.05) where developing (8.4% compared to 1.2%) and transitional economy countries (5.2% compared to 3.2%) were more often concerned (*p* < 0.001). In 2017 included trials were more often funded by pharmaceutical companiesor industry (*p* < 0.001).

Table 2 shows changes in important measures of methodological quality and reporting.

As compared to 2012, we observed an improvement in 2017 in the reporting of the presence of a data monitoring committee (42.0% compared to 34.4%; *p* < 0.001). As compared to 2012, there was a positive change in registering trials in trial registries in 2017 and, among clinical trial registries, the clinicaltrials.gov database had increased popularity (registration rate in clinicaltrials.gov was: 78.4% compared to 68.9%; *p* = 0.03). Also, significantly more RCTs reported sample size calculation (60.4% compared to 49.6%; *p* < 0.01) in 2017 as compared to 2012. Although fewer RCTs specified plan to collect adverse effects in 2017 (48.4% compared to 74%; *p* < 0.001), they reported harms more often in 2017 (68%compared to 52%; *p* < 0.001). When we investigated the reporting of results, we observed that the number of RCTs with statistically significant results of the primary outcome was lower in the 2017 sample (69.2% compared to 78.8%; *p* < 0.01). Further, there were more publications with neutral conclusions in 2017 (18.4% compared to 7.2%; *p* < 0.01). There were no statistically significant differences between 2012 and 2017 in the number of intentions to treat analyses, in the type of outcomes (as most outcomes were objective), or specific types of primary outcomes.

### 3.2. Rısk of Bias Assessment

We provided a risk of bias assessment by each domain for trials published in 2012 and 2017 year (Table 3). Compared with 2012, more 2017 RCTs were rated low (70.4% compared to 38.8%) and fewer were rated unclear (20.4% compared to 50%; *p* < 0.001) risk for allocation concealment. Fewer 2017 RCTs were rated low (50.8% compared to 65.6%; *p* < 0.001) risk for blinding of participants and personnel, for blinding of outcome assessors (82.4% compared to 90.8%; *p* < 0.001), and selective outcome reporting (62.8% compared to 80.0%; *p* < 0.001). A similar proportion of 2017 RCTs were rated low risk for random sequence generation (59.6% compared to 56.0%), and for incomplete outcome data (74% compared to 73.6%;) compared to 2012. In 2017, more RCTs were rated low (42.8% compared to 33.6%) risk for other risk of bias (*p* < 0.01). More trials were rated low (29.2% compared to 21.2%) for overall risk of bias in 2017 compared to 2012 (*p* < 0.01).

In 2017, multicenter trials (OR 0.39, 95% CI 0.18 to 0.80), drug trials (OR 0.53, 95% CI 0.29 to 0.97), and registered trials (OR 0.06, 95% CI 0.003 to 0.31) were also more likely to have a low overall RoB. In 2012, there was not yet a significant difference between multicenter or single-center trials (OR 0.52, 95% CI 0.24 to 1.22), drug trials, and non-drug trials (OR 0.82, 95% CI 0.44 to 1.56). Trial registration was not yet shown to have positive effects on RoB in 2012 either (OR 0.85, 95% CI 0.38 to 1.84).

## 4. Discussion

### 4.1. Summary of Main Findings

We observedthat randomized cardiovascular clinical trials changed significantly from 2012 to 2017 years in several characteristics related to their study design and reporting. Respectively, RCTs in the 2017 sample were published mostly in specialty cardiovascular journalswith a higher number of authors from Asia and Europe compared to trials published in 2012. The 2017 sample includedmore parallel trialsevaluating drug interventions. The number of industry-funded clinical trials increased from 2012 to 2017, while the number of trials funded by the academy decreased. In the 2017 sample, more trials were from developing and transitional economy countries. Multinational trials had a significantly higher proportion in the 2017 sample compared with 2012.

As compared to 2012, in 2017 there were significant changes in important measures of methodological quality and reporting, including an improvement in the reporting of the presence of a data monitoring committee, and a positive tendency of registering trials in trial registries. Also, we observed that significantly more RCTs reported sample size calculations in 2017 as compared to 2012.

We also observed notable changes over five years in the ability of randomized cardiovascular research trials to properly estimate the true intervention effect. The 2017 trials were more likely to have a low RoB than 2012 for overallRoBs. However, the 5-year change was not clearly in the direction of improvement, as we observed a lower number of RCTs with alow RoB for blinding of participants and personnel and blinding of outcome assessors in 2017 as compared to 2012. In 2017, multicenter trials, drug trials, and registered trials were also more likely to have a low overall RoB than single-center, non-drug, non-registered trials. In 2012, these RoB differences were not yet present between RCTs with specific characteristics.

### 4.2. Strengths and Weaknesses of the Study

For both investigated publication years we selected our samplerandomlyfrom Cochrane CENTRAL as themost comprehensive resource of RCTs. The samples covered areas of the prevention, diagnosis, and treatment of cardiovascular diseases, including acute myocardial infarction, heart failure, arrhythmia, coronary revascularization, and chronic coronary artery disease. Most of our trials were registered in clinical trial registries, so essential trial details were double-checkedin both the full-text article andthe registry. We used the most accurate tool for risk of bias (methodologicalquality) assessment including RCTs. Two independent reviewers performed data extraction and RoB assessment, discrepancies were always resolved by discussion.

This study has also some limitations. Our sampleincludedabout 10% from all eligible cardiovascular disease trials published in the years 2012 and 2017 only in the English language. This study was not pre-registered witha detailed statisticalanalysis plan. We have chosen cardiovascular trials with participants aged 18 years or older, therefore our results are not applicable to pediatric trials in cardiovascular medicine.

As we started our research in the year 2018 and intended to evaluate changes over time, we decided to investigate publications from 2017, and from five years earlier, from 2012. However, since 2017, the publication characteristics may have changed further.It also has to be emphasized that the 2017 sample differed from the 2012 sample in many study design and reporting features, which may have impacted our RoB results.Although we have collected supplementary information from trial registries and published protocols, this was not detailed enough to compare information across these different information sources.

### 4.3. Discussion of Findings Considering Other Studies

The risk of bias in CVD RCTs generally decreased over the 5 years. This is consistent with the conclusions of Vinkers et al. [20], who reported significant improvement in the level of risk of bias of RCTs over the past years in connection with increased knowledge about mandatory trial registration and journal requirements.

Our study revealed that trial registration positively influenced RoB.This finding is in line with prior researchinvestigating clinical trial registration and the risk of bias. A study among clinical trials included in Cochrane systematic reviews of interventions published between 2014 and 2019 found that clinical trial registration was associated with a low risk of bias for all bias domains examined except for attrition bias, and for overall risk of bias [21]. Registered trials were at lower risk of overall bias than non-registered trials in Latin America and the Caribbean [22]. Prospectively registered trials had a significantly lower risk of bias compared to unregistered trials across all domains of health research [23].We found that multi-center trials were more likely at low risk of bias than single-center trials. This could be associated with that multi-center studies allow for better control of study quality than single-center studies [24]. Our findings areconsistent with Tamborska et al., who found that RCTs at lower risk of bias were more likely to use multicenter recruitment in neurology trials [25].

Our investigation has shown that drug trials had a more favorableimpact on RoB than non-drug trials, which might be related tothe strict regulations these pharmaceuticaltrials must follow.Similarly, Cho Y et al. found that most drug trials were at low risk of bias for blinding participants and personnel, while almost two-thirds of non-drug trials were at high risk of bias for blinding participants and personnel in cardiopulmonary resuscitation and emergency cardiovascular care [26].

Existing differences and the positive beneficial impact of regulations can be observed in the planning phase of trials when regulated clinical trialprotocols were described to follow reporting guidelines to a greater extent than non-regulated trials [27].

### 4.4. Implication for Practice and Future Research

We observed some improvements with respect to some important study design features and some specific RoB domains over 5 years. However, there were also some methodological features and RoB domains thatchanged in an unfavorabledirection or remained unchanged. This points to the need to continue to pay close attention to the planning and conduct of RCTs in the field of cardiovascular clinical research.

This study identified several features of clinical trial planning and conducting that need further improvement in the field of cardiovascular research. Improvementsin study design, conduct, and reporting will decrease research waste and support the realization of evidence-based decisions in the field of cardiology. Journal adoption of existing reporting guidelines may lead to potential mechanisms to ensure improvements in overall clinical trialquality. We would emphasize that a paper that adheres to reporting guidelines better places a clinical decision-maker to assess the quality of the trial design and conduct and to interpret its findings accurately, improving the potential of the research to be impactful and meaningful to patients and clinical practice.

## 5. Conclusions

We call cardiovascular disease researchers to try to avoid possible risks of bias duringplanning and conducting their cardiovascular RCTs, and followreporting guidelines when communicating their results to ensure the validity of trial results and their effective translation to evidence-based cardiovascular patient care.

## Figures and Tables

**Table 1:** 2012 (*n* = 250) and 2017 (*n* = 250).

Characteristics	2012, *n* (%)	2017, *n* (%)	*p* Value
Type of Journal			<0.001
Specialty cardiovascular journal	96 (38.4%)	100 (40.0%)	
General cardiovascular journal	41 (16.4%)	46 (18.4%)	
Specialty medical journal	26 (10.4%)	49 (19.6%)	
General medical journal	50 (20.0%)	41 (16.4%)	
Other	37 (14.8%)	14 (5.6%)	
Continent of corresponding author			<0.05
Africa	3 (1.2%)	0 (0.0%)	
Asia	57 (22.8%)	65 (26.0%)	
Australia	10 (4.0%)	2 (0.8%)	
Europe (excluding UK)	70 (28.0%)	93 (37.2%)	
North America	89 (35.6%)	69 (27.6%)	
South America	8 (3.2%)	13 (5.2%)	
United Kingdom	13 (5.2%)	8 (3.2%)	
Total	250 (100%)	250 (100%)	
Study type			0.093
Efficacy/Superiority	244 (97.6%)	237 (94.8%)	
Equivalence	2 (0.8%)	3 (1.2%)	
Non-inferiority	4 (1.6%)	4 (1.6%)	
None of the above	0 (0.0%)	6 (2.4%)	
Study design			<0.01
Cluster	7 (2.8%)	0 (0.0%)	
Parallel	201 (80.4%)	231 (92.4%)	
Crossover	34 (13.6%)	15 (6.0%)	
Factorial	5 (2.0%)	4 (1.6%)	
Other	3 (1.2%)	0 (0.0%)	
Intervention type			<0.001
Alternative therapeutic	24 (9.6%)	32 (12.8%)	
Behavioral	0 (0.0%)	2 (0.8%)	
Cell therapy	0 (0.0%)	1 (0.4%)	
Communication, organizational, or educational	4 (1.6%)	13 (5.2%)	
Device	17 (6.8%)	23 (9.2%)	
Diet, nutrition	26 (10.4%)	10 (4.0%)	
Drug	117 (46.8%)	139 (55.6%)	
Prevention or screening	43 (17.2%)	20 (8.0%)	
Rehabilitation or psychosocial	18 (7.2%)	6 (2.4%)	
Surgery or radiotherapy	1 (0.4%)	3 (1.2%)	
Other	0 (0.0%)	1 (0.4%)	
Type of control			0.628
Active intervention	153 (61.2%)	160 (64.0%)	
No intervention	10 (4.0%)	21 (8.4%)	
Placebo	86 (34.4%)	68 (27.2%)	
Other	1 (0.4%)	1 (0.4%)	
Was the study multicenter?			0.063
Yes	117 (46.8%)	157 (62.8%)	
No	131 (52.4%)	93 (37.2%)	
Unclear	2 (0.8%)	0 (0.0%)	
Was the study multinational?			<0.05
Yes	45 (18.0%)	69 (27.6%)	
No	205 (82.0%)	181 (72.4%)	
Where were participants recruited from?			<0.001
Developing country	3 (1.2%)	21 (8.4%)	
Transitional country	8 (3.2%)	13 (5.2%)	
Established market economy	239 (95.6%)	216 (86.4%)	
	250 (100%)	250 (100%)	
Who funded the study?			<0.001
Academic or Research institute	113 (45.2%)	94 (37.6%)	
Government	44 (17.6%)	24 (9.6%)	
Industry for device	4 (1.6%)	10 (4.0%)	
No external funding	3 (1.2%)	4 (1.6%)	
Pharmaceutical	36 (14.4%)	48 (19.2%)	
Private	13 (5.2%)	50 (20.0%)	
Unclear	37 (14.8%)	21 (8.4%)	
Total	250 (100%)	250 (100%)	
How was the study population selected?			0.775
Inpatients	144 (57.6%)	133 (53.2%)	
Outpatients	98 (39.2%)	116 (46.4%)	
Unclear	7 (2.8%)	1 (0.4%)	
Primary diagnostic category in the study			0.971
Circulatory system	250 (100%)	244 (97.6%)	
Congenital malformations	0 (0.0%)	1 (0.4%)	
Factors influencing health status	0 (0.0%)	2 (0.8%)	
Metabolic disease	0 (0.0%)	2 (0.8%)	
Unclear	0 (0.0%)	1 (0.4%)	

Footnote: Intervention categories were defined based on Wood et al., 2008 [18] Multicenter trials were defined as trials with two or more administratively distinct study centers. Multinational applied to the countries from which patients were enrolled. The economic status of the country was defined based on Panagiotou et al., 2013 [19].

**Table 2:** Changes in important measures of methodological quality and reporting.

Study Characteristics	2012, *n* (%)	2017, *n* (%)	*p*
Funding source			0.002
Specified	243 (97.2%)	229 (91.6%)	
Not specified	7 (2.8%)	21 (8.4%)	
Consent obtained			0.895
Reported	250 (100%)	248 (99.2%)	
Not reported	0 (0.0%)	2 (0.8%)	
Number of patients approached to participate in the study			0.854
Reported	2 (0.2%)	12 (4.8%)	
Not reported	248 (99.8%)	238 (95.2%)	
Number of patients consented to participate in the study			0.534
Reported	2 (0.2%)	12 (4.8%)	
Not reported	248 (99.8%)	238 (95.2%)	
Number of participants randomized			0.972
Reported	2 (0.2%)	2 (99.8%)	
Not reported	248 (99.8%)	248 (2.0%)	
Number of participants analyzed			0.887
Reported	2 (0.2%)	1 (0.4%)	
Not reported	248 (99.8%)	249 (99.6%)	
Sample size calculation			<0.01
Reported	124 (49.6%)	151 (60.4%)	
Not reported	126 (50.4%)	99 (39.6%)	
Data Monitoring Committee			<0.001
Yes	86 (34.4%)	105 (42.0%)	
No	39 (15.6%)	94 (37.6%)	
Unclear	125 (50.0%)	51 (20.4%)	
Analysis described as intention to treat			0.120
Yes	232 (92.8%)	222 (88.8%)	
No	18 (7.2%)	28 (11.2%)	
Primary outcome specified in trial registry			0.823
Yes	135 (54.0%)	157 (62.8%)	
No	115 (46.0%)	93 (37.2%)	
Primary outcome was objective			0.652
Objective	247 (98.8%)	248 (99.2%)	
Subjective	3 (1.2%)	2 (0.8%)	
Type of primary outcome			0.124
Behavioural	20 (8.0%)	6 (2.4%)	
Biomarker	40 (16.0%)	21 (8.4%)	
Physiological	172 (68.8%)	206 (82.4%)	
Psychological	5 (2.0%)	5 (2.0%)	
Techniques/Training	8 (3.2%)	6 (2.4%)	
Quality of life	3 (1.2%)	1 (0.4%)	
Other	2 (0.8%)	3 (1.2%)	
At least one statistically significant outcome			0.899
Yes	213 (85.2%)	215 (86.0%)	
No	37 (14.8%)	35 (14.0%)	
Significant statistical primary outcome			<0.01
Yes	197 (78.8%)	173 (69.2%)	
No	53 (21.2%)	77 (30.8%)	
The author’s overall conclusion			<0.01
Negative	32 (12.8%)	34 (13.6%)	
Neutral	18 (7.2%)	46 (18.4%)	
Positive	193 (77.2%)	170 (68.0%)	
Insufficient evidence (intermediate)	7 (2.8%)	(0.0%)	
Planning to collect adverse effects/events or side effects			<0.001
Reported	185 (74.0%)	121 (48.4%)	
Not reported	65 (26.0%)	129 (51.6%)	
Harms reported			<0.001
Yes	130 (52.0%)	170 (68.0%)	
No	120 (48.0%)	80 (32.0%)	
Blinding performed			0.087
Yes	126 (50.4%)	145 (58.0%)	
No	124 (49.6%)	105 (42.0%)	
Trial registered			0.238
Yes	135 (54.0%)	192 (76.8%)	
No	115 (46.0%)	58 (23.24%)	
Primary register			0.031
clinicaltrials.gov	124 (68.9%)	164 (78.4%)	
Other	56 (31.1%)	45 (21.6%)	
Primary outcome stated the same in trial registry and in the publication			<0.001
Yes	132 (52.8%)	183 (73.2%)	
No	76 (30.4%)	26 (10.4%)	
N/A	42 (16.8%)	41 (16.4%)	

The behavioral outcome included attitudes and specific (e.g., eating) behaviors; biomarkers were defined as markers measured as an indicator of biologicalor pathogenic processes or pharmacologic responses to an intervention;physiological outcomesr eflected how a patient feels, functions or survives; psychological and quality of life outcomes included different scales measuring these variables.We used ’no’ when something hasn’t been done when it could have been possible; and used ‘N/A’ when it doesn’t apply to that particular trial.

**Table 3:** Risk of bias assessments by domain in 2012 (*n* = 250) and in 2017 (*n* = 250).

RoB Domains	*N* (%) in 2012	*N* (%) in 2017	*p*
**Random sequence generation**			
Low	140 (56.0%)	149 (59.6%)	0.381
Unclear	95 (38.0%)	68 (27.2%)	
High	15 (6.0%)	33 (13.2%)	
**Allocation concealment**			
Low	97 (38.8%)	175 (70.0%)	<0.001
Unclear	125 (50.0%)	51 (20.4%)	
High	28 (11.2%)	24 (9.6%)	
**Blinding participants and personnel**			
Low	164 (65.6%)	127 (50.8%)	<0.001
Unclear	73 (29.2%)	112 (44.8%)	
High	13 (5.2%)	11 (4.4%)	
**Blinding outcome assessors**			
Low	227 (90.8%)	206 (82.4%)	<0.001
Unclear	19 (7.6%)	33 (13.2%)	
High	4 (1.6%)	11 (4.4%)	
**Incomplete outcome data**			
Low	184 (73.6%)	185 (74.0%)	0.469
Unclear	60 (24.0%)	57 (22.8%)	
High	6 (2.4%)	8 (3.2%)	
**Selective outcome reporting**			
Low	200 (80.0%)	157 (62.8%)	<0.001
Unclear	48 (19.2.0%)	67 (26.8%)	
High	2 (0.8%)	26 (10.4%)	
**Other bias**			
Low	84 (33.6%)	108 (42.8%)	<0.01
Unclear	131 (52.4%)	106 (42.4%)	
High	35 (14.0%)	36 (14.4%)	
**Overall bias**			
Low	53 (21.2%)	73 (29.2%)	<0.01
Unclear	142 (56.8%)	99 (39.6%)	
High	55 (22.0%)	78 (31.2%)	

## Data Availability

The raw data supporting the conclusions of this article will be made available from the corresponding author without undue reservation.
